# Sleep Disturbances and Disorders in Children and Adolescents with Cerebral Palsy: A Narrative Review

**DOI:** 10.3390/jcm14217828

**Published:** 2025-11-04

**Authors:** Rebecca A. Rausch, Caroline Miller, Amelia Hensler, Mark G. Goetting, Dilip R. Patel

**Affiliations:** 1 Homer Stryker M.D. School of Medicine, Western Michigan University, Kalamazoo, MI 49008, USAdilip.patel@wmed.edu (D.R.P.); 2 Doctoral Program in Clinical Psychology, School of Psychology, Fielding Graduate University, Santa Barbara, CA 93105, USA

**Keywords:** cerebral palsy, children, adolescents, sleep disturbances, sleep disorders

## Abstract

**Background/Objectives:** Children and adolescents with cerebral palsy experience multiple associated comorbidities or impairments that impact their day-to-day life and psychosocial functioning. Sleep difficulties, disturbances, or disorders are a widely recognized concern for youth with cerebral palsy. We aimed to provide an updated narrative review of the most recent research on sleep disturbances or disorders in youth with cerebral palsy. **Methods**: A search of the literature cited in the PubMed database published between August of 2019 and August of 2024 was completed, and relevant articles were reviewed. **Results**: Relevant areas included the prevalence and type of sleep concerns seen in children and adolescents with cerebral palsy in addition to the relationship with sleep concerns and behavior, pain, comorbidities, physical activity, quality of life, and impact on caregivers. Sleep disturbances and disorders occur at a higher frequency in children and adolescents with cerebral palsy with adverse impact on their quality of life. Sleep concerns appear to be associated with several associated concerns. **Conclusions**: Sleep disturbances occur at a higher frequency in youth with cerebral palsy and are associated with a wide range of conditions, symptoms, and impact on quality of life. Treatment recommendations are in line with typically developing children or children with neurodevelopmental disorders. Future directions for research are identified.

## 1. Introduction

Cerebral palsy is a group of disorders affecting movement, posture, and muscle tone (these factors affect sleep positioning, arousal, and pain) resulting from a static brain injury or lesion that occurred in the prenatal or perinatal time period [1]. It is the most common cause of physical disability in children, with a prevalence of 1.5 to 3 per 1000 live births, which varies between high- and low-income regions [1,2,3]. Preterm birth is the most significant risk factor for cerebral palsy [1]. Based on the findings on physical examination, the clinical phenotypes of cerebral palsy are generally categorized as spastic diplegia, spastic quadriplegia, spastic hemiplegia, extrapyramidal (dyskinetic), and ataxic [2,4]. Motor impairments are the hallmark of CP, and individuals with this condition also experience multiple associated and secondary or acquired conditions that can significantly impact psychosocial quality of life [2,5,6].

Capturing the prevalence of sleep concerns or disorders in youth with cerebral palsy is difficult due to age-dependent variance in sleep habits and needs, even among typically developing children. Because of this, estimates of prevalence range widely. A 2006 study [7] found that 44% of children with CP experienced at least one sleep disorder, with 23% noted to have a pathological total sleep score compared to 5% of the general population. Newer studies have noted higher rates, with a 2021 study noting 72.2% of children with CP to have at least one sleep problem compared to 46.4% of typically developing children [8]. While these numbers differ substantially, the prevalence of sleep disorders in youth with CP is consistently higher than in the general population. While most studies have focused on sleep duration, research has indicated that other aspects of sleep are affected in children with cerebral palsy. Sanguino et al. reported an average of 14 nighttime awakenings in children with cerebral palsy compared to 4 typical of quality sleep [9]. It may also be difficult to capture the scope of sleep disorders in children with CP, as the types of sleep problems that youth with CP experience are highly variable, including difficulty initiating and maintaining sleep, sleep–wake transitions disorders, disorders of arousal, and sleep-related breathing concerns [10]. Different subtypes of cerebral palsy and Gross Motor Function Classification System (GMFCS) level affect the frequency and type of sleep disturbance. For example, higher GMFCS levels are associated with an increased number of sleep problems, in particular early awakening and difficulty maintaining sleep [8,9].

Sleep difficulties in children and adolescents with CP may be caused or further exacerbated by comorbid conditions such as pain, motor impairment, incontinence, and mental conditions; however, such a relationship has also been proposed to be reciprocal, contributing to worsened behavior, mood, and motor dysfunction [7,9,11].

Sleep disturbances or disorders may have a significant impact on the quality of life of children and adolescents with CP. One in three children in a sample of preschool- and school-aged children with CP had impaired health-related quality of life scores, and those with sleep problems had greater than seven times the odds of having impairment in psychosocial health-related quality of life [12]. Regardless of the prevalence or type of sleep problem, parents report that there is insufficient screening or assessment of sleep problems, as well as inadequate knowledge on the causes and management of those problems in children with CP [13,14].

Due to the relatively recent increase in awareness of the importance of sleep management in the care of children with CP, there is a lack of interventions that have been specifically studied in this population. With this increase in awareness of the importance of sleep and associated research, there is a need to summarize current research on sleep concerns in children with CP. The aim of this paper is to review recent research on sleep concerns or disorders in children with CP, highlighting research published in the last five years.

## 2. Materials and Methods

We conducted a literature search focused on the most recent five years of research (August of 2019 through August of 2024) focused on the sleep of children with CP. This PubMed search included Mesh terms relevant to CP, sleep, and sleep disorders within children and adolescents. Based on this search, 31 articles were included for review. The specific search used was as follows:

(“Cerebral Palsy”[Mesh] OR “cerebral palsy”[ti] OR “early brain lesion”[ti] OR “perinatal stroke”[ti] OR “hemiplegia”[ti] OR “diplegic”[ti] OR “dyskinetic cerebral palsy”[ti]) AND (“Child”[Mesh] OR “Adolescent”[Mesh] OR child*[ti] OR adolescent*[ti]) AND (“Sleep”[Mesh] OR “Sleep–Wake Disorders”[Mesh] OR sleep[ti] OR “sleep disorder*”[ti] OR “sleep disturbance*”[ti]).

The inclusion criteria for selection were as follows: 1. research studies focused on children with CP and 2. studies focused on sleep concerns or disorders. We excluded studies based on the following criteria: 1. average age of participants set to 18 or older, or data from participants not collected from the child, adolescent, or youth age range (0–18 years old); 2. editorials or comments; 3. systematic reviews that did not include a meta-analysis; and 4. case studies. All articles were selected by the first author, a doctoral-level psychologist, utilizing the inclusion and exclusion criteria for selection.

**Figure 1:**
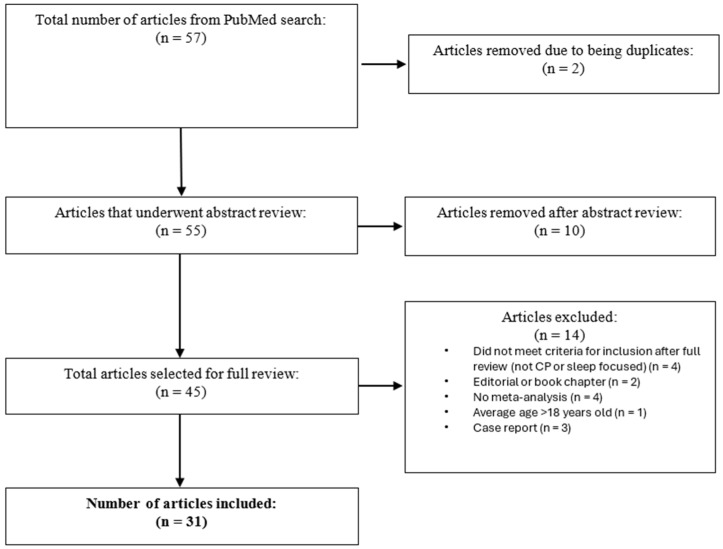
Flow chart of article selection.

Figure 1 shows the flow chart of article selection.

The aim of this paper is to review selected research on sleep concerns or disorders in children with CP, highlighting research published in the most recent five years. Articles included in this paper are shown within Table 1.

## 3. Results

### 3.1. Prevalence of Sleep Problems

Many studies included in this review reported the frequency of sleep disturbances in their sample of children and adolescents with CP [8,10,15,16,18,20,22,23,25,26,28,29,31,37,39]. Despite this, capturing accurate prevalence rates of sleep disturbances for this population is difficult. A 2019 meta-analysis of 23 studies found a pooled sleep disturbance prevalence rate of 23.4%, as measured by the Sleep Disturbance Scale for Children (SDSC) [16]. However, individual studies have reported sleep problems in as many as 81% of their specific samples [22], suggesting substantial variability in frequency due to factors like comorbidity and vulnerability to sampling errors. Additionally, inconsistency across studies in terms of methodological approaches further complicates reporting of prevalence rates. Many studies utilized self-reports of sleep symptoms and concerns. Parent or caregiver reports were more common than self-reports. Differences in methodologies, ranging from differences in assessments used, reporters, and samples, likely significantly impacted the differences in prevalence rates between studies and the wide range of reported sleep concerns for this population.

Furthermore, differences in sleep needs across age and developmental stages complicate comparisons, as sleep standards and types of sleep problems vary widely between younger children and adolescents. While some studies reviewed included participants ranging in age from infancy to adulthood [22,28], others included participants in two or more developmental stages [8,15,23,25,31,37]. Studies that include broad age ranges may not fully account for these developmental differences, leading to inconsistent prevalence estimates between studies. Nearly two-thirds of children with CP may not be meeting age-appropriate sleep recommendations [34]. However, specific age was not a consistent predictor of sleep concerns, with Horwood et al. [16] reporting that sleep problems were more prevalent in older children and adolescents than younger children, while other studies reported that age did not predict sleep disturbances [20,31] or that sleep disturbances were more common in younger children [25]. This variability may again be attributed to factors like comorbidity, but the lack of consistency does demonstrate the need for continued evaluation for the impact of age on sleep disturbances.

Despite these inconsistencies, studies overwhelmingly conclude that sleep disturbances are significantly more prevalent in children with CP compared to their typically developing peers [8,16,31].

### 3.2. Assessment of Sleep Concerns

Studies included in this review frequently examined specific sleep concerns using the SDSC. One study focused on the Sleep Questionnaire for Children with Severe Psychomotor Impairment (SNAKE). The SNAKE is a caregiver proxy report measure specifically tailored to children with significant psychomotor impairments and the included scales are matched to the International Classification of Sleep Disorders-2 [21]. Caregiver reports were consistently used across studies.

Wrist and forehead actigraphy have been shown to be a valid tool for assessment of sleep for children with CP. In a 2022 study [27], actigraphy and concurrent polysomnography was obtained in 13 children with CP and 13 children without CP for one night of sleep, and then continued wrist actigraphy measurement was taken in the home environment for one week. The authors developed a novel weighted logistic regression sleep–wake scoring method for actigraphy and reported high agreement with polysomnography variables and improved performance (in specificity and accuracy) [27]. It is important to note for both clinicians and researchers that there can be substantial discrepancies between subjective and objective measures of sleep used for children with CP. van Rijssen et al. [35] measured the sleep of 38 children with CP (GMFCS I–III) for one week using a sleep diary and two objective/device-based measures of sleep, which included actigraphy and a bed sensor. Except for the measurement of total time in bed, poor agreement was found between the three measurement approaches. Some agreement was found between the sleep diary and bed sensor for total sleep time and wakefulness after sleep onset (for weekend nights measured). Based on these results, van Rijssen and colleagues [35] noted the promise of bed sensor measurement for children with CP, acknowledging that further research is needed to confirm feasibility. Additionally, authors recommended against the use of actigraphy alone for measurement or diagnosis of sleep concerns in children with CP, instead recommending use of both subjective and device-based measurement of sleep for this population [35].

### 3.3. Specific Sleep Concerns

In addition to describing how children with CP experience sleep disturbances generally, researchers have investigated the specific sleep concerns experienced by this population. One study compared the sleep experiences of children with CP, ages 0–11, to an age- and gender-matched group of typically developing peers and found that children with CP experienced more frequent sleep problems in all domains measured, including difficulty falling asleep, waking through the night, waking too early in the morning, and struggles with daytime fatigue [8]. These experiences were even more acute for children with CP who were non-ambulatory.

One investigation of children ages 6–18 found a particular relationship between severe GMFCS levels and disorders of initiating and maintaining sleep (DIMSs), as well as a correlation between gross motor function and disorders of arousal (DAs) and sleep–wake transition disorders (SWTDs) [23]. Highlighting the variability in sleep disturbances experienced by this population, Samota et al. [31] reported the most common sleep disturbance in their sample was DA, though DIMSs, SWTDs, and disorders of excessive somnolence (DOESs) were also significantly experienced. In a questionnaire-based observational study, ref. [10], of the participants identified with sleep disturbance as measured by the SDSC, 78.2% endorsed items consistent with a DIMS, 44.4% endorsed items consistent with a DA, and 44.4% endorsed items consistent with a SWTD.

Wolter and colleagues [32] reported that moderate to severe obstructive sleep apnea was found in 49% of their sample of children referred for sleep disordered breathing who underwent polysomnography. Additionally, in a sample of individuals with CP undergoing intrathecal baclofen therapy (primarily at GMFCS IV or V), one or more nighttime symptoms of sleep-related breathing disorders were found in more than 82% of participants [24]. Periodic limb movements were found at higher rates in children with CP. Retrospectively examining polysomnography with leg electromyography in children with either neuromuscular disease or CP, an elevated periodic limb movement index was observed in 9.6% of participants with CP [41]. Research over the past 5 years highlights that children with CP exhibit significant variability in the type of sleep concerns that they experience.

### 3.4. Sleep and Behavior

Three studies included in this review specifically investigated the relationship between sleep disturbances and behavioral outcomes in children with CP. All studies reviewed found that sleep disturbances are significantly associated with behavior problems in this population [15,22,39]. One study found that disturbances in sleep were the most influential predictor of the frequency and severity of self-injurious behavior, aggressive/destructive behavior, and stereotyped behavior in children with CP ages 2.5–18.5 [22]. Whittingham et al. [39] reported that behavioral problems uniquely explained over 20% of variance in sleep problems in children ages 8–12. The relationship between sleep and behavior is likely bidirectional, such that poor sleep may lead to poor behavior, but problems with behavior may also lead to problems with sleep [39].

Another study found that children ages 4–12 with significant sleep disturbances were seven times more likely to have clinically significant behavioral problems, particularly in the areas of conduct problems, emotional symptoms, hyperactivity, and issues with peers [15]. The same study found that sleep was the only significant predictor of these behavioral concerns when controlling for comorbidities [15].

### 3.5. Sleep and Pain

The experience of physical pain or discomfort is a common comorbidity in children with CP and is particularly impactful on sleep. Children with mobility limitations may be unable to physically adjust their position if they become uncomfortable during the night, leading to sleep arousal and distress. As such, contemporary research has sought to more clearly understand the impact of pain on sleep and its relationship with sleep disturbances. In this review, most studies that assessed pain found that the experience of nighttime pain was associated with increased sleep disturbances [17,20,22,37,39].

In one study examining pain in youth with CP [17], chronic pain was reported (through mixture of self-report and proxy-report) by 31.4% of participants, and 2 in 3 children reported acute pain. Nearly 25% of children experiencing acute pain reported that their pain often or almost always interfered with their sleep, and 47.1% of children with chronic pain reported that their pain often or almost always interfered with their sleep [17]. Nighttime pain has been found to be significantly associated with sleep problems, and this relationship may be more acute for children with dyskinetic CP and more severe motor function impairment (GMFCS level V) [20]. Within one sample of children ages 8–18 who experienced moderate to severe pain had worsening sleep problems over time, and improvement in pain led to improvement in sleep [37]. Interestingly, child perception of pain improvement also resulted in improved perception of sleep quality, even if their pain was reported to be severe at onset [37], suggesting that improvement in sleep can be achieved even if the experience of pain cannot be fully eliminated.

One investigation by Lang et al. [25] of children ages 4–14 found that the presence of pain was not associated with sleep disturbances at all. This disparate finding may be attributed to the use of parent-reported measures, which may not have accurately captured the child’s experience of pain [25].

### 3.6. Sleep and Epilepsy

Epilepsy was the most frequently examined comorbid condition in this review. A 2019 meta-analysis revealed that children with comorbid epilepsy experienced more sleep problems than children without this condition [16]. Other research has found similar results [26,31]; for example, Whittingham et al.’s [39] study found that epilepsy increased the odds of a child experiencing sleep disturbances by 16 times. Epilepsy was not found to be a risk factor for sleep problems within one sample (although involuntary muscles contractions were) [28] or to be associated with sleep disturbance in another sample [25].

Of note, one study found a significant association between sleep disturbance and medication use, highlighting that medications aimed at associated impairments or comorbidities (such as those used for epilepsy) may impact youth’s quality of sleep [26].

### 3.7. Sleep and Visual Impairment

The study by Lang et al. [25] found that visual impairments, measured as a dichotomous variable, were significantly associated with sleep disturbances, and Dreier et al. [21] found that disturbances remaining asleep correlated with severe visual and cognitive impairment. The meta-analysis by Horwood et al. [16] also demonstrated that children with comorbid visual, auditory, or cognitive impairments had more sleep disturbances than children without these comorbidities. However, the study by Whittingham et al. [39] found that visual impairments were not associated with sleep disturbances, though the presence of ‘mild impairments’ was included in the analysis. Again, these inconsistencies may be attributed to differences in methodology.

### 3.8. Other Associated Factors, Conditions, and Sleep Problems

The presence and severity of gross motor function have also been examined. The contemporary literature finds that more severe motor and mobility impairment, classified as GMFCS levels IV and V, is strongly associated with sleep disturbances [8,16,20,23,28,31,39]. Additional studies have found that sleep disturbances are reported at all levels, such that specific GMFCS-based classification is not uniquely associated with sleep problems [18], or that GMFCS levels can only predict certain types of sleep disturbances but not sleep problems overall [23]. However, Dreier and colleagues [21] reported that sleep behavior in a sample of 100 children with CP was primarily related to greater functional impairment, as measured by GMFCS or Bimanual Fine Motor Function, over CP type or comorbidities. The level of functional independence also appears to be a factor associated with sleep in children with CP, with lower functional independence levels being associated with greater sleep problems in this population [33]. Inconsistent consideration of CP subtypes and mobility levels across studies makes it difficult to draw definitive conclusions about the overall prevalence of sleep disturbances in this population.

Comorbidity of CP and autism spectrum disorder has been shown to be associated with a higher number of sleep problems when compared to children with either autism spectrum disorder or CP alone [29].

A final study specifically investigated how the comorbid presence of gastrointestinal (GI) symptoms impacts sleep for this population. In their study of children ages 2.5–18.5 (M = 6.88), Leader et al. [22] found that the presence of GI symptoms, like abdominal pain and constipation, was significantly associated with sleep disturbances, likely due to the discomfort associated with these symptoms.

### 3.9. Sleep and Physical Activity

The relationship between physical activity and sleep has also been examined in children with CP, with mixed results. Smit et al. [19] reported that within a sample of children with CP, participants were meeting recommended duration of sleep, and 67% of children and 20% of adolescents slept more than the recommended number hours of sleep on weekends. Interestingly, they found no correlation between duration of physical activity and sleep duration. However, the authors reported a moderate negative correlation between sedentary time and self-rated sleep quality [19].

Exploring the impact of an intensive rehabilitation program on sleep problems, Kim et al. [36] recruited 36 children with developmental delays, 19 (59.3%) of whom had CP. The rehabilitation program included physical and occupational treatment sessions that lasted for 30 min and was either admission-based (twice-a-day sessions >20 times per week for two months) or outpatient sessions (once-a-day sessions >10 times per week for two months). Sessions were dependent on the child’s needs and could include treatments focused on speech, sensory integration, or cognitive concerns. Significant improvements were reported in DIMSs and disorder of excessive somnolence sub-scale scores of the SCDC for children with CP. No improvements were reported for the total score of the SCDC or other sub-scores [36].

After monitoring the physical activity and sedentary behavior of children with CP between the ages of 3 and 12 years old, Gerritsen et al. [40] reported that the relationship between sleep and physical activity in children with cerebral palsy does not appear to be consistent with what is seen in typically developing children without cerebral palsy. Higher levels of light or moderate-to-vigorous physical activity were negatively associated with sleep efficiency and total amount of sleep the following night, while sedentary behavior/time was positively associated with total amount of sleep and sleep efficiency the following night in their sample. These results were inconsistent with initial hypotheses, and the authors highlight that these outcomes point to the need to further investigate the complex relationship between physical activity, sedentary behavior, and sleep in children with CP, particularly with longitudinal designs [40].

### 3.10. Sleep and Quality of Life

Quality of life has been found to be associated with sleep concerns or disorders in children with CP. A study by Samota et al. [31] found that for children ages 4–12, the presence of sleep disturbances was significantly negatively correlated with quality of life, such that more frequent and severe disturbances in sleep negatively impacted the physical and emotional dimensions of quality of life. This finding was true for both breathing-related sleep disturbances and the presence of other types of sleep disturbances [31]. These results were mirrored by Badaru and colleague’s [26] study, which also highlighted the association between sleep and quality of life.

Another study of children ages 8–12 investigating sleep problems, and various possible contributing factors thereof, included quality-of-life measures in their analysis [39]. This study found that higher perceptions of pain was related to a higher reported pain-related quality of life, demonstrating that the experience of pain alone may not directly lead to diminished quality of life for this population [39]. This divergent finding highlights the need for further research on how sleep quality influences broader aspects of life for children with CP.

### 3.11. Effects on Caregivers

In addition to the impact on quality of life for children with CP, sleep problems also negatively impact their caregivers [13]. A common theme when interviewing parents or caregivers of children with CP about their children’s sleep is the need for constant vigilance [13,14]. Parents or caregivers report waking frequently or sleeping less in order to monitor their child’s breathing and pain, to reposition them, and sometimes just to check on them [13,14]. Some parents have even characterized their disturbances in sleep as akin to “water torture”, with effects on daytime functioning and family dynamics [13].

A study by Hulst et al. [8] compared sleep deprivation and sleep satisfaction between caregivers of children with CP and typically developing children and found no significant difference between groups, with the only exception being those caregivers whose child was non-ambulatory tended to endorse more sleep deprivation. Another study found that caregivers whose children have sleep disturbances were more likely to have sleep problems themselves but that caregiver sleep was otherwise not associated with gross motor function impairment [18].

A study by Lang et al. [25] specifically examined the relationship between psychological outcomes and sleep quality for caregivers of children with CP. Caregiver sleep quality was significantly associated with various aspects of psychological health (anxiety, depression, stress, and wellbeing) and that child sleep problems, specifically their need for nighttime support, impacted caregiver sleep quality [25]. This finding is inconsistent with a study by Obrecht et al. [23] which demonstrated that the need for nocturnal support was not associated with poorer sleep quality in caregivers, even for children with significant gross motor function impairment [23].

Recent qualitative studies focused on parental perspectives highlight the significant impact and burden put on families in managing their children’s sleep concerns [14]. All parents interviewed reported concerns and needs in caring for their child’s sleep, both in the home environment and in healthcare settings. Parents expressed concerns about their children’s safety or wellbeing during sleeping hours, leading to monitoring their child’s sleep and discussion of worries related to long-term consequences of sleep difficulties both on their children and themselves. Interviews indicated that parents felt that sleep was infrequently asked about during their child’s medical appointments and that providers did not have knowledge of sleep concerns relevant to their child. Interviews also led to the identification of needs for these families, including areas of social support, information on sleep concerns and solutions, greater focus on sleep during healthcare interactions, and an intervention that would be helpful for their child [14].

### 3.12. Sleep Interventions

#### 3.12.1. Sleep Systems

Sleep systems, which are supports used in lying for individuals with severe motor disorders, have also been explored for children with CP [30]. Examined over the course of 5 months (following a 1-month baseline), sleep systems were implemented for four children with CP at GMFCS level V. The authors reported either improvement in sleep quality or quality equal to that found during baseline. The experience of pain remained unchanged following implementation of the sleep system, but individualized caregiver goals had a statistical improvement. Due to the small sample size and subjective data collection (self-report), further research is needed to fully understand the impact of sleep systems for this population [30].

#### 3.12.2. Melatonin

A double-blind clinical trial conducted in 2024 [38] examined the impact of melatonin in children with CP. Children with CP between the ages of 2 and 12 years old with complaints of sleep disorders were randomly assigned to a melatonin or placebo group with a treatment duration of one month. Sleep variables were measured by actigraphy via a wearable smart watch. The authors reported a significant effect of melatonin on sleep disorders for children with CP. The greatest improvement observed in the intervention group was found for the duration of time until participants fell asleep, with the average time until sleep initiation being 6.6 min in the intervention group and 39.3 min in the placebo group. Both groups demonstrated improvement in average length of sleep, with an increase from 218 min to 274 min in the intervention group and from 243 to 251 min in the control group. The authors noted that improvement in sleep depth and duration for those in the melatonin group differed significantly from the control group [38].

#### 3.12.3. Adenotonsillectomy

Wolter et al. [32] reported that within a sample of children with CP referred for sleep disordered breathing, improvements in obstructive apnea–hypopnea index scores were found after adenotonsillectomy. However, children with CP (when compared to children without CP who also underwent adenotonsillectomy) demonstrated more postoperative complications (43.5% of children with CP compared to 8.7% of children without CP) in addition to greater odds of respiratory complications. Increased respiratory complications were found in children at GMFCS level V and those with swallowing impairments [32].

## 4. Discussion

This review highlights research published in the last five years focused on sleep in youth with CP. The studies reviewed highlight a trend of increasing attention to the impact of child sleep disturbances across several domains, including behavior, experiences of pain, and secondary outcomes such as caregiver sleep and health. Compared to typically developing peers, youth with CP are likely to experience a higher prevalence of sleep disturbances [8,16,31]. Sleep is essential for the overall health and wellbeing of children [42], and inadequate sleep is associated with a variety of negative consequences for adolescents [43]. Sleep concerns early in life may also have consequences for future functioning. Childhood sleep has a neurocognitive impact, with implications for intelligence, memory, attention, and academic performance [44]. Based on animal studies, sleep deprivation may lead to neurobehavioral impairments, which are particularly concerning should they occur during early developmental processes [45]. Although further research is needed, recent studies support that sleep plays an important role in brain development [46]. Early childhood sleep concerns may be part of a causal pathway to mental/behavioral health concerns in adolescence, particularly for anxiety, depression, and attention deficit hyperactivity disorder [47]. Although these relationships have not explicitly been studied within children with CP, the heightened risk of childhood sleep concerns for this population puts them even further at risk for detrimental health and developmental outcomes.

Of the comorbidities associated with sleep concerns discussed in this review, epilepsy was most frequently examined. The somewhat inconsistent findings across all studies reviewed may be partially explained by differences in methodologies. The degree to which researchers examined or accounted for the presence of anticonvulsant medication, the use of parent-reported versus self-reported questionnaires measuring sleep disturbances, the degree to which the participants epileptic condition was considered medically controlled, and the presence of impaired motor functioning all varied between studies. However, compared to healthy peers, children with epilepsy experience a variety of sleep disturbances or disruptions [48]. Epilepsy can affect sleep through nocturnal seizures and medication side effects, some of which can worsen gastroesophageal reflux, insomnia, and sleep apnea. Further, insufficient sleep and sleep fragmentation lowers the seizure threshold and promotes subclinical and apparent seizures. In this bidirectional relationship between sleep and seizures, sleep disorders, seizures, or antiepileptics can further trigger seizures [49]. Importantly, epilepsy often greatly heightens parental anxiety because of the possibility of status epilepticus and sudden unexpected death [50]. Most families have emergency medication and a protocol already in place but worry whether they will detect this emergency, adding to the vigilance for parents. Future research should seek to better understand if and how the comorbid presence of epilepsy contributes to sleep disturbances for the pediatric CP population specifically.

Additionally, sleep disturbances and the experience of pain or discomfort secondary to physical impairment due to CP (or a related comorbidity) have also emerged as a topic of recent attention. Youth with chronic pain associated with a variety of chronic illnesses or conditions experience sleep disturbances [51]. While most [17,20,22,37,39], but not all [25], studies reviewed found that the experience of nighttime pain was associated with sleep disturbances, future investigations into the relationship between pain and sleep disturbances should investigate the longitudinal impact of pain on sleep and other domains [37]. In a systematic review on the prevalence and characteristics of pain in children and young adults with CP [52], prevalence rates of endorsed pain ranged from 14% to 76% of participants with CP between the ages of 2 and 23 years old. Pain was most frequently reported in the lower limbs, indicating a possible pain target for this population [52]. The studies included in our review assessed for pain generally, but children with CP may have a variety of causes of pain [53]. In adolescents with chronic pain, sleep disruption may be a stronger predictor of pain the next day when compared to the experience of pain being a predictor of sleep the next night [54] and it could be important to examine this relationship for youth with CP.

Within this review, epilepsy, pain, visual impairment, and more were associated with impairment in sleep for this population. Comorbidity or multimorbidity is common in individuals with CP. Compared to the general population, individuals with CP experience higher rates of neurological, mental, and medical disorders [55]. This is true for concerns considered to be co-causal with or a complication of CP, in addition to comorbidities not assumed to be a complication of CP [55]. With consideration of higher rates of comorbidities, we may see an overall increased risk for sleep concerns in individuals with CP related either to CP itself or to the presence of these comorbid concerns or diagnoses and how these concerns interact with one another to impact sleep. For example, gastroesophageal reflux is common in children with CP and affects sleep with pain and regurgitation [56]. Or the experience of pain may contribute to greater sleep impairment, which could then be associated with a higher frequency of seizures, which could then be associated with worse sleep.

Three studies examined treatment approaches for sleep concerns [30,32,38]. However, behavioral sleep interventions are often a recommended treatment option for children and adolescents with sleep disorders or disturbances [57,58]. With the consideration of high rates of sleep concerns in the pediatric CP population, efforts should be made to provide children and their families with treatment options aimed at improving sleep overall. While sleep concerns clearly impact youth with CP, little research has been conducted on behavioral or psychological sleep interventions specific to this population. There is a need for studies examining the effectiveness of behavioral or nonpharmaceutical interventions for youth with CP [59]. Caregiver-implemented interventions researched for typically developing children have been recommended for children with or at high risk of CP under the age of two [60]. International practice guidelines for children 0–2 years of age either with CP or at risk of CP recommend parental education and behavioral interventions aimed at establishing positive sleep hygiene approaches, with the acknowledgement that most intervention research in this area has focused on neurodevelopmental disorders other than CP [61]. Indeed, behavioral sleep interventions demonstrate an improvement in sleep for children with neurological and neurodevelopmental disorders [62], and a recent umbrella review and meta-analysis on nonpharmacological interventions for sleep disturbances demonstrated that behavioral interventions for children and adolescents (from a variety of clinical populations) had positive impacts on multiple sleep concerns [63].

Studies on sleep hygiene typically incorporate behavioral and environmental components. Behavioral components focus on physical activity, stimulus control, naps, bedtime routine, food intake, caffeine/alcohol consumption, and timing of sleep, while environmental components focus on the bedroom environment, including factors like noise, temperature, light, and bedding [64]. Stimulus control approaches focus on altering the association between an individual’s bed and sleep or wakefulness [64]. Parental sleep education programs are fundamental in the management of sleep difficulties in youth [65] and have been associated with improvement in sleep onset delay for children with autism spectrum disorder [66]. Behavioral interventions for child and adolescent sleep concerns are often multicomponent, incorporating parent training, psychoeducation on sleep, and specific sleep interventions or cognitive–behavioral therapy approaches [63]. Specific interventions often include some form of extinction (unmodified or graduated), positive routines, bedtime fading, scheduled awakenings [65], and reinforcement [67]. Further research should emphasize the evaluation of these specific sleep interventions with the pediatric CP population to ensure applicability and effectiveness.

Some children with CP require noxious, even painful, interventions for their care. This may include suctioning, injections, and ROM stretching. The caregiver will at times provide loving comfort and at other times inflict discomfort, even pain. The child will not know what will happen when that caregiver enters the room. One suggested strategy is for the parents and home nurses to always wear a specific-colored gown or cap when entering the room for unpleasant procedures. A mask is perhaps a better option because it hides facial expression. They could leave the room promptly and return without a mask to give comfort.

The current review highlighted gaps in the literature focused on sleep concerns in youth with CP. While high rates of sleep concerns have been identified across samples of children with CP, there was substantial variability in methods utilized to identify prevalence rates, leading to very wide ranges of prevalence rates. While caregiver and parental perspectives are extremely valuable when understanding the experience of families with CP, overreliance on parental self-report data may have influenced reported prevalence rates. A scoping review of sleep assessments for children with severe CP recommended the use of both the SDSC and actigraphy to assess sleep [68]. Future studies utilizing self-report (ideally of youth with CP themselves in addition to caregivers) and a more objective measurement of sleep, such as actigraphy, would allow for a clearer understanding of the actual prevalence rates of sleep concerns within this population.

While evidence-based sleep interventions exist for typically developing children or children with developmental disabilities, little research has been conducted on the application of these interventions to the pediatric CP population specifically. Future research should emphasize the evaluation of established interventions to determine the appropriateness for children with CP and to identify any factors or approaches that may need to be tailored to this population. Additionally, pain was identified as a factor that often impacts sleep. A novel area of future research could be on the impact of treating the experience of pain for youth with CP and observing the subsequent impact on sleep. Future research should also explore the directionality of pain and sleep to better understand if and how sleep disturbance may exacerbate comorbidities contributing to pain and vice versa.

The three studies that explored interactions between sleep and behavior demonstrated a clear relationship between the presence sleep disturbances and poor behavioral outcomes in children with CP [15,22,39]. In the general population, sleep problems at 18 months of age have been shown to be predictive of later emotional and behavioral problems [69]. Additionally, there is support for the idea that a reciprocal relationship exists between sleep problems and behavior problems [70]. Sleep duration has also been found to be associated with cognitive functioning in healthy children, specifically that shorter duration of sleep in in children is associated with poorer cognitive functioning and academic performance [71]. Future research should clarify the causal connection between sleep and behavior concerns for the pediatric CP population, particularly with young children and adolescents. Additionally, future research should explore how sleep and behavior impact child functioning in other domains like academic performance [15,39].

There are limitations to the current review. Our abstract and article search included articles published within PubMed only, which could have limited the diversity and comprehensiveness of the articles included in this review. Abstract review and article selection was completed by the first author, a doctoral-level psychologist. We acknowledge that this author’s professional background or personal perspective may have influenced which articles were included in the current review. Adherence to the inclusion and exclusion criteria was utilized in attempt to limit the impact of bias in selection of articles. Additionally, we excluded reviews that did not include a meta-analysis. Our aim was to highlight the most recent research evidence available on the sleep of children with CP. By excluding reviews without a meta-analysis, we may have limited novel or valuable interpretations on this subject.

## 5. Conclusions

Sleep can be a particular challenge for youth with CP and can be exacerbated by mobility limitations and comorbid conditions requiring nocturnal support. Contemporary research exploring sleep disturbances in this population examines various interactions between sleep problems and behavior, pain, comorbidities, physical activity, quality of life, and the secondary impact on caregivers. All studies reviewed on sleep relied heavily on caregiver reports and, to a much lesser extent, child self-report scales. Few of the studies reviewed used an objective measure of sleep. Future investigations of the intersection between CP and sleep should integrate these objective measures. Treatment recommendations for sleep disturbance or disorders for children with CP are typically in line with typically developing children or children with neurodevelopmental disorders [60,61]. Future research should directly examine outcomes of behavioral interventions on sleep disturbance specifically for youth with CP.

## Figures and Tables

**Table 1:** Selected articles included in review.

Author/Year	Purpose	Study Design	Sample	Relevant Findings
Horwood, Li, Mok, Oskoui, Shevell, & Constantin (2019) [15]	Determine the prevalence of behavioral challenges in preschool- and school-aged children with CP and assess the association between behavioral challenges and sleep problems, nighttime pain, and child characteristics.	Cross-Sectional	Caregivers of 113 children with CP	Approximately 26% of children with CP had behavioral challenges. Sleep problems and nighttime pain were also associated with behavioral challenges. Sleep and behavioral problems were highly associated even when adjusting for nighttime pain and age.
Horwood, Li, Mok, Shevell, & Constantin (2019) [16]	Review and conduct a meta-analysis of the literature to determine the prevalence of sleep problems in children with CP, including prevalence in various subgroups.	Meta-Analysis	23 English or French studies included, representing 2908 children with CP	Older children with CP had higher rates of sleep problems than younger children; children with more severe CP phenotype had more sleep problems than children with milder phenotype; and children with CP and additional comorbidities such as epilepsy and auditory, visual, and cognitive impairments had more sleep problems than children without comorbidities. The effects of comorbidities on sleep were not consistent across all studies reviewed.
Ostojic, Paget, Kyriagis, & Morrow (2020) [17]	Determine the prevalence, impact, and management of pain in children with CP.	Cross-Sectional	280 children with CP between the ages of 5 and 18 years old and their caregivers	Acute pain was reported by 67.1% of participants and chronic pain was reported by 31.4% of participants. Pain frequently interferes with sleep.
Petersen, Francis, Reddihough, Lima, Harvey, & Newall (2020) [18]	Determine the frequency and type of sleep problems for children with CP with their parents. Explore whether and from whom parents seek help for sleep concerns. Determine whether the parents who sought help found the advice/treatment effective.	Population-Based Cohort (Online) Survey	126 parents/caregivers of children with CP	Sleep is a problem for half the cohort studied. Parents of children with CP who have sleep problems are more likely to have problems with sleep themselves. GMFCS level does not seem to be related to sleep problems (problems were reported across all levels). Help for sleep problems is not always effective (effective ~30% of the time).
Smit, Zwinkels, Takken, Hulst, de Groot, Lankhorst., & Verschuren (2020) [19]	Explore the relationship between sleep quantity, sleep quality, physical activity, and sedentary behavior in children with CP, in addition to assessing their sleep quantity.	Cross-Sectional	36 children with spastic CP with an average age of 15 years old	Sedentary behavior is correlated with sleep quantity in children with CP. Children with CP in this sample were getting the recommended duration of sleep.
Löwing, Gyllensvärd, & Tedroff (2020) [20]	Describe and explore insomnia in children with CP ages 5–10.	Explorative Cross-Sectional Design	118 children with CP included in first part of study (medical records review); 95 parents/caregivers of the original 118 participated in second part of study (pre-structured telephone interview)	Insomnia was present to a high extent in this cohort. Sleep problems were reported across all subtypes and GMFCS levels but were most common in level V and those with dyskinetic CP. Sleep problems were moderately associated with the presence of seizures. Pain was highly correlated with sleep problems. A relationship could exist between sleep problems and motor limitations/GMFCS levels.
Hulst, Gorter, Voorman, Kolk, Van Der Vossen, Visser-Meily, Ketelaar, Pillen, & Verschuren (2021) [8]	Describe the frequency and type of parent-reported sleep problems. Describe parent-reported satisfaction with their sleep and child sleep. Describe child factors related to sleep problems. Compare sleep outcomes between typically developing children and those with CP.	Multicenter Cross-Sectional Study with Comparison Group	90 children with CP and their parents; comparison group included 157 typically developing children and their parents	Children with CP were more likely to have sleep problems than typically developing children (72.2% vs. 46.4%), and these problems are more significant for non-ambulatory children. These problems more significantly impact daily functioning for children with CP. These problems result in less parental satisfaction in their child’s sleep. No significant difference between parents of children with CP and parents of typically developing children in their own sleep satisfaction or sleep deprivation. Non-ambulatory children with CP also experience greater impairment in daily functioning related to sleep problems than ambulatory children with CP.
Kulkarni & Jadhav (2021) [10]	Examine prevalence and pattern of sleep disorders in children with CP.	Questionnaire-Based Observational Study	200 children with CP between the ages of 1 and 14 years old; children with health problems involving cardiorespiratory system, gastroesophageal reflux, epilepsy, or children taking anticonvulsant medications were excluded	Of the sample, 62% of had a pathological total sleep score on the Sleep Disturbance Scale for Children. Disorders of initiating and maintaining sleep occurred in 78.2% of participants who had a pathological sleep score. Those with GMFCS V and with quadriplegia were impacted most severely.
Dreier, Kapanci, Lonnemann, Koch-Hogrebe, Wiethoff-Ubrig, Rauchenzauner, Blankenburg, Zernikow, Wager, & Rostasy (2021) [21]	Explore sleep of children with CP. Considering the impact motor impairment and comorbidities, explore potential sleep problems in children with different CP subtypes.	Hospital-Based, Prospective Study	100 children with CP between the ages of 2 and 18 years old	Associations were found between gross motor functioning and fine motor functioning and CP comorbidities. Functional impairment (as measured by gross or fine motor functioning) is more crucial for sleep concerns than CP or CP subtype.
Leader, Molina Bonilla, Naughton, Maher, Casburn, Arndt, & Mannion (2021) [22]	Identify frequency of GI symptoms, sleep problems, internalizing/externalizing symptoms, and ASD symptoms in a sample of children with CP. Examine impact of comorbidities on frequency and severity of behavior problems in this sample.	Parent-Completed Questionnaires	104 youth with CP	A high frequency of behavior problems, sleep problems, gastrointestinal symptoms, ASD symptoms, and internalizing/externalizing symptoms was found in this sample. Relationships were found between the following: sleep problems and behavior problems; GI symptoms and sleep problems; and GI symptoms and internalizing/externalizing problems. Behavior problems were predicted by sleep problems, internalizing/externalizing symptoms, ID, and ASD symptoms.
Petersen, Reddihough, Lima, Harvey, & Newall (2021) [13]	Examine the impact/experience of sleep disturbance in addition to the experience of seeking sleep solutions or treatment for caregivers of children with CP.	Semi-Structured Qualitative Parent Interviews with Thematic Analysis	19 parents of children with CP	Sleep is often not a significant priority for caregivers or health professionals due to other health concerns. Parents experience difficulties and challenges in finding effective sleep interventions for their children with CP. Children’s needs are often different from what parents are provided by systems and services.
Obrecht, Fischer de Almeida, Maltauro, Leite da Silva, Bueno Zonta, & de Souza Crippa (2021) [23]	Investigated the influence of gross motor function impairment on sleep disturbances of children with CP, their need for nocturnal support, and caregiver quality of sleep.	Cross-Sectional	87 children with CP	A total of 52% of children had inadequate scores in at least one factor of a sleep measure, 64% of children were taking meds that had the potential to interfere with sleep, and 44.8% of children received nocturnal support from caregivers. Children with greater gross motor impairment needed more nocturnal support. Among caregivers, 62.1% were found to have poor sleep quality.
Mohon, Sawyer, Pickett, Bothwell, Brinton, Sobremonte-King, & DelRosso (2021) [24]	Determine prevalence of sleep-related breathing disorders in children with CP who are receiving intrathecal baclofen therapy.	Retrospective Chart Review	87 children with CP. Medical records were reviewed from the Children’s Hospital Colorado and Seattle Children’s Hospital between January 1st, 1995, and December 31st, 2019. Participants had to have been receiving intrathecal baclofen therapy and had at least one nocturnal polysomnography performed.	Over 82% of participants had one or more nighttime symptoms as measured on a sleep-related breathing disorder screening questionnaire. There was no worsening or improvement in sleep-related breathing disorder symptoms.
Lang, Boucaut, Guppy, & Johnston (2021) [25]	Examine relationship between sleep problems in children with CP, caregiver sleep, and caregiver psychological health.	Cross-Sectional	94 caregivers of children with CP	Poor sleep quality was reported in 71% of caregivers. Sleep problems were reported in 51% of children. Children’s sleep problems and their need for nighttime attention contributed to parental sleep quality. The factor most associated with need for nighttime support was motor impairment. Caregiver sleep quality was significantly associated with their psychological health.
Badaru, Hassan, Ahmad, Nuhu, & Lawal (2021) [26]	Examine the prevalence of sleep disturbance in children with CP and assess the impact of sleep disturbance on exercise participation and quality of life.	Cross-Sectional	200 children with CP and 200 siblings of children with CP	A total of 31.5% of children with CP had a sleep disturbance, and these children suffered more sleep disturbance than their siblings. This sleep disturbance had a negative impact on these children’s quality of life in addition to a negative impact on their ability to participate in exercises both at home and in a clinic-based setting.
Xue, Licis, Boyd, Hoyt, & Ju (2022) [27]	Validate the use of actigraphy in children with CP and assess their sleep patterns.	Cross-Sectional Cohort Study	13 children with CP and 13 children without CP between the ages of 2 and 17 years old	Actigraphy was shown to be a valid tool for assessing sleep in children with CP. Sleep efficiency and duration were worse in children with CP. Author-developed algorithms demonstrated increased specificity and accuracy compared to existing algorithms.
Zh Chia, Tan, Yeo, Teoh, & Min Ng (2022) [28]	Describe sleep problems in a local population of children with CP.	Cross-Sectional	151 participants with CP ages 1–24 (median age = 6.18)	Of the participants, 46% had difficulty with one or more aspects of sleep. GMFCS level V and involuntary muscle contractions were significant factors in sleep problems.
Leader, Mooney, Chen, Whelan, Naughton, Maher, & Mannion (2022) [29]	Examined frequency of comorbidities in children with ASD alone, CP alone, and those with comorbid CP and ASD.	Screening Study	96 children with a diagnosis of CP, ASD, or comorbid CP and ASD	Significant group differences in sleep problems, social communication, and adaptive behavior. ID significantly predicted levels of adaptive behavior.
Hulst, Voorman, Pillen, Ketelaar, Visser-Meily, & Verschuren (2022) [14]	Explore the perspective of parents regarding sleep care for their children with CP.	Inductive Thematic Analysis of Semi-Structured Interviews	18 parents of children with CP between the ages of 1 and 8 years old. Four of the parents had a child above the age of 8 years old and were asked to reflect on the time their child was between 1 and 8 years old	All parents interviewed expressed that they had a range of concerns or needs when caring for the sleep of their child with CP. Parents expressed concerns about caring for their child during both the day and night, perceived difficulties or deficiencies in healthcare, and had limited knowledge of or attention to sleep concerns from healthcare professionals. The authors called for a wakeup call to these parent-identified concerns and shortcomings in healthcare.
Wood & Brown (2022) [30]	Examine the impact of sleep systems on sleep quality/quantity and pain for youth with CP, in addition to examining the outcome for caregivers.	Exploratory Study	4 children with CP (average age of 11.5 years old)	While pain levels remained unchanged following introduction of the sleep system, sleep quantity either improved or stayed the same as baseline.
Samota, Singh, Aggarwal, & Malhotra (2023) [31]	Assess the relationship between sleep disorders and quality of life in children with CP.	Cross-Sectional	117 children with CP and additional control group (n = 117) of age- and gender-matched children without CP	Sleep disorders were more prevalent in children with CP compared to controls, and this experience of a sleep disorder was associated with a decreased quality of life for children with CP.
Wolter, Scheffler, Li, End, McKinnon, Narang, Amin, Chiang, Matava, & Propst (2023) [32]	Assess outcomes of adenotonsillectomy for obstructive sleep apnea in children with CP.	Retrospective Chart Review	97 children with CP were assessed for sleep disordered breathing, and 74 underwent polysomnography. Perioperative data was available in 23 children with CP, and these children were compared to 23 age-matched children without CP.	The obstructive apnea–hypopnea index was improved for children with CP following adenotonsillectomy, but children with CP had higher rates of post-adenotonsillectomy complications.
Gunaydin & Tuncer (2023) [33]	Examine the impact of level of functional independence on sleep and constipation in children with CP.	Cross-Sectional Observational Study	60 children with CP between the ages of 4 and 18 years old	Lower levels of functional independence were associated with worse sleep and symptoms of constipation in children with CP.
Hulst, Gorter, Obeid, Voorman, van Rijssen, Gerritsen, Visser-Meily, Pillen, & Verschuren (2023) [34]	Using actigraphy, describe and measure 24 h activities of children with CP, and examine adherence to the 24 h activity guidelines.	Cross-Sectional Observational Study	54 children with CP aged between 3 and 12 years old	Adherence to the 24 h guidelines was low. In total, 35% of the sample met age-appropriate sleep duration recommendations. Only 5.9% of children met the combined 24 h guidelines for both physical activity and sleep.
van Rijssen, Hulst, Gorter, Gerritsen, Visser-Meily, Dudink, Voorman, Pillen, & Verschuren (2023) [35]	Examine how device-based and subjective assessments of sleep relate or compare to one another in children with CP.	Cross-Sectional Observational Study	38 children with CP aged between 2 and 12 years old	Poor agreement was found between sleep diaries, actigraphy, and a bed sensor on many aspects measured. Actigraphy only had satisfactory agreement for total time in bed with the bed sensor and sleep diaries. The bed sensor only had satisfactory agreement for total time in bed and total sleep time with the sleep diaries. With consideration of these discrepancies, the authors recommended using both subjective and device-based measures to assess sleep in children with CP.
Kim, Jung, Chang, & Park (2024) [36]	Examine the effect of an intensive rehabilitation program on sleep problems in children with developmental delays.	Prospective Design	36 children with developmental delays, 19 of which had CP	The intensive rehabilitation program was associated with significant improvement in difficulties initiating and maintaining sleep; however, no significant improvement in other areas, as measured by the Sleep Disturbance Scale for Children, was found.
Shearer, Côté, Hogg-Johnson, & Fehlings (2024) [37]	Prospectively measure the association between pain intensity trajectory and sleep disturbance.	Cohort study	89 children with CP	An association between pain intensity trajectory and increased sleep problems was only supported for those with the most severe pain, who reported the most significant sleep disturbance. Children with decreasing pain had less perceived sleep disturbance, even if their pain level was higher at the start. Children with moderate to severe pain had greater sleep disturbance, which also worsened with time.
Goldouzi, Akhondian, Beiraghi Toosi, Mehrad Majd, Shekari, & Babaei (2024) [38]	Examine the effect of melatonin on sleep disorders in children with CP.	Double-blind clinical trial	50 children with CP between the ages of 2 and 12 years old randomly assigned to the melatonin or control group	A significant effect of melatonin on sleep disorders was reported for this sample. The most significant effect of melatonin was on the duration of time to fall asleep, and melatonin was also associated with an increased sleep duration.
Whittingham, Benfer, Sakzewski, Wotherspoon, Burgess, Comans, Keramat, Ware, & Boyd (2024) [39]	To study sleep problems in population sample of children with CP.	Cross-Sectional	86 children with CP	Sleep problems in children with CP are common, with 44% of participants in the clinical range for disordered sleep. Sleep problems were associated with pain-related quality of life, child behavior, and epilepsy.
Gerritsen, Hulst, van Rijssen, Obeid, Pillen, Gorter, & Verschuren (2024) [40]	Examine the bidirectional and temporal relationship between physical activity and sleep in children with CP who are ambulatory.	Cross-Sectional	51 children with CP between the ages of 3 and 12 years old	Children with CP who are ambulatory may not sleep better after physical activity. This relationship is complex in children with CP and should be further investigated.
Nisbet, Davey, & Nixon (2024) [41]	Assess the prevalence of periodic limb movements in children with CP or neuromuscular diseases.	Retrospective Review and Analysis	239 children, 114 of which had CP and 125 had neuromuscular diseases	Elevated periodic limb movement index was reported at a higher prevalence in children with CP or a neuromuscular disease, at 9.6% in each group.

## Data Availability

No new data were created or analyzed in this study. Data sharing is not applicable to this article.

## Abbreviations

The following abbreviations are used in this manuscript:

CP	Cerebral Palsy
GMFCS	Gross Motor Function Classification System
SDSC	Sleep Disturbance Scale for Children
DIMS	Disorder of Initiating and Maintaining Sleep
DA	Disorder of Arousal
SWTD	Sleep–Wake Transition Disorder
SNAKE	Sleep Questionnaire for Children with Severe Psychomotor Impairment
